# Preparation and application of specific chicken yolk antibodies in detecting *Brucella*

**DOI:** 10.3389/fvets.2025.1552097

**Published:** 2025-05-12

**Authors:** Xinru Qi, Shiqi Zhao, Qianhan Huang, Yun Wang, Qichuan Pei, Yixiao Chen, Dehui Yin, Tiansong Zhan

**Affiliations:** ^1^Jiangsu Engineering Research Center of Biological Data Mining and Healthcare Transformation, Xuzhou Medical University, Xuzhou, Jiangsu, China; ^2^Department of Dermatology, The Affiliated Huai'an Hospital of Xuzhou Medical University, The Second People's Hospital of Huai'an, Huai'an, China; ^3^Key Laboratory of Human Genetics and Environmental Medicine, Xuzhou Medical University, Xuzhou, Jiangsu, China

**Keywords:** *Brucella*, detection, chicken yolk antibody, ELISA, fusion protein

## Abstract

Brucellosis, caused by *Brucella*, is a severe zoonotic disease. Conventional IgG antibody-based ELISA testing faces challenges such as false positives and cross-reactivity. In this study, three specific chicken yolk antibodies (IgY) targeting *Brucella* were isolated from the eggs of immunized hens, and a method for detecting *Brucella* utilizing these antibodies was developed and subsequently compared to traditional IgG antibodies. These IgY antibodies were generated against a fusion protein, LPS, and whole-cell antigen, and their potency was evaluated through indirect ELISAs. Testing was conducted to assess cross-reactivity, limit of detection, and detection in simulated samples. The IgY antibodies demonstrated high potency and no cross-reactivity with common foodborne pathogens. Both LPS-IgY and *Brucella*-IgY showed excellent detection capabilities in identifying *Brucella*, particularly in food samples. These results underscore the potential of using LPS-IgY and *Brucella*-IgY antibodies as replacements for conventional IgG antibodies in *Brucella* detection, especially in the realm of food safety. The implications of this study are significant, as it presents a promising alternative approach for detecting *Brucella* in food products, thereby reducing the risk of transmission and ensuring public health and safety.

## 1 Introduction

Brucellosis, caused by *Brucella*, is a globally prevalent zoonotic disease that poses a significant threat to both human and animal health (1–4). It annually causes over 2.1 million infections worldwide (5), leading to substantial economic losses in the livestock industry due to its impact on animal health and mortality (6). While China has seen a shift from widespread *Brucella* outbreaks before the 1980s to scattered distribution, recent years have shown an increase in incidence, and more than 70,000 new human brucellosis cases reported in 2023 (7).

Foodborne transmission remains a primary route for *Brucella* infection (8). Infected animals carry *Brucella* in their bodily fluids and tissues, and humans and animals can contract the infection by consuming contaminated animal products, such as raw milk, meat, and dairy products that have not been adequately heated or processed (5, 9). The risk of foodborne brucellosis transmission has heightened due to the extended food supply chain and increased international trade.

The detection of *Brucella* involves pathogenic, molecular biology, and immunological approaches. While isolation culture is considered the gold standard, its implementation is hindered by technical complexity, high cost, and the risk of infection for personnel conducting the tests, making it challenging for practical application (10). Molecular biology techniques, such as polymerase chain reaction (PCR) and loop-mediated isothermal amplification technology (LAMP), offer high-throughput and rapid detection of *Brucella* by targeting pathogen DNA. Although these methods demonstrate excellent limit of detection, accuracy, and fast detection, they require specialized equipment, which may not be readily available in grassroots laboratories (11, 12). Immunological assays for *Brucella*, including tests like fluorescence polarization assay (FPA), enzyme-linked immunosorbent test (ELISA), and colloidal gold immunochromatography assay (GICA) (13, 14), offer simplicity, sensitivity, and rapid results. However, these methods often suffer from a high false positive rate and cross-reactivity with other bacteria due to the lack of specific antibodies or antigens. Current commercial kits require the preparation of *Brucella* monoclonal antibodies, which involves a significant investment of time, labor, and resources.

Chicken yolk antibody (IgY), a highly conserved relative of immunoglobulin G (IgG), has shown promising benefits and a favorable safety profile, particularly in animal models for infectious diseases in humans. IgY offers distinct advantages compared to mammalian IgG, as IgY antibodies are more readily available for use in diagnostic assays, with lower background levels and reduced cross-reactivity than IgG commonly used in antibody production (15). IgY demonstrates rapid action, ease of production, and cost-effectiveness. Through the utilization of laying hens, significant quantities of IgY antibodies can be efficiently generated with minimal environmental impact and infrastructure requirements (16). The aim of this study is to develop an indirect ELISA based on *Brucella* IgY and assess its effectiveness using food-simulated samples. These samples, which consist of milk and meat matrices spiked with known concentrations of *Brucella*, are designed to simulate real-world contamination scenarios and offer a practical assessment of the performance of IgY antibodies in detecting *Brucella* within complex food matrices. This methodology is essential for comprehending the potential applications of IgY antibodies in the domains of food safety and public health. Furthermore, the limit of detection and cross-reactivity of *Brucella*-specific IgY antibodies will be compared with IgG antibodies. This research sets the groundwork for the advancement of novel immunological assays for *Brucella* detection.

## 2 Materials and methods

### 2.1 Materials

Fusion protein (17) (contains the sequences of the outer membrane proteins Omp16, Omp25, Omp31, Omp2b, and BP26, containing 22 validated epitopes) is preserved by our laboratory. *Brucella abortus S66* (*B. abortus S66*, a reference strain), *Salmonella* (ATCC 13311, *Salmonella enterica* subsp. *enterica* serovar Typhimurium), *Escherichia coli* O157: H7 (*E. coli* O157: H7) (ATCC 35350), *Listeria monocytogenes* (*L. monocytogenes*) (ATCC 19111), *Staphylococcus aureus* (*S. aureus*) (ATCC 25923), *Ochrobactrum anthropi* (*O. anthropi*) (ATCC 49188), IgG aganist *B. abortus* (purified from bovine) and LPS (extracted and purified from *B. abortus*) were provided by China Animal Health and Epidemiology Center (Qingdao, China), all the details for the preparation of antigens are in the Supplementary material S1. Specific pathogen-free (SPF) laying hens were purchased from Shandong Haotai Laboratory Animal Breeding Ltd. (Jinan, China). HRP Conjugated Rabbit Anti-Bovine IgG and HRP Conjugated Goat Anti-Chicken IgG were purchased from Life Technologies Corp. (USA).

### 2.2 Immunization of chickens

Eight SPF laying hens (Leghorn aged 25 weeks) were allocated into three experimental groups, labeled 1A, 1B, 2A, 2B, 3A, and 3B. A negative control group received PBS (0.01M, pH 7.4) solvent. The first set of experiments (1A, 1B) involved immunizing each chicken with 10^6^ CFU/mL of killed *B. abortus* S66 suspended in an equal volume of Freund's adjuvant. In the second group (2A, 2B) and third group (3A, 3B) of experiments, chickens were inoculated with 2 mg/mL of laboratory-preserved fusion proteins and 2 mg/mL of LPS respectively, each mixed with Freund's adjuvant. The immunizations were carried out by intramuscular injection and administered every 2 weeks for a total of 5 rounds, with 300 μL per injection. Freund's complete adjuvant (FCA) was used for the initial immunization, while Freund's incomplete adjuvant (FIA) was used for subsequent immunizations. The titer of IgY antibodies in the chicken serum and yolk was assessed on days 14, 28, 42, 56, and 70 post-immunization.

### 2.3 Purification and identification of IgY

Fourteen days after the last immunization, eggs were collected continuously for 1 week. IgY was extracted from egg yolks using the PEG6000 method (18) as follows:

(a) The collected eggs were sterilized with 75 % ethanol. The shells were then gently cracked, the egg whites removed, and the yolks collected using a yolk-white separator commonly used in home kitchens. The yolks were placed on filter paper and gently swirled to remove excess egg white. Next, the yolks were punctured, and the yolk liquid was collected in a 50 mL centrifuge tube. The volume of the collected yolk liquid (V1, mL) was recorded.
(b) To the collected yolk liquid, 2 × V1 (mL) of PBS was added, and the total volume (V2, mL) was recorded. Then, 3.5 % (w/v) PEG6000 (amount of PEG6000, g = 3.5 % × V2) was added. The mixture was gently rotated for 10 min and mixed well. Centrifuge at 4°C for 20 min at 10,000 × g. The supernatant was filtered through filter paper, collected, and the volume (V3, mL) was recorded.
(c) To the collected supernatant, 8.5 % PEG6000 (amount of PEG6000, *g* = 8.5 % × V3) was added. The mixture was gently rotated for 10 min and mixed well. Centrifuge at 4°C for 20 min at 10,000 × g, discard the supernatant, and collect the precipitate.
(d) The precipitate was resuspended in 10 mL PBS, and 1.2 g PEG6000 was added. The mixture was gently rotated for 10 min and mixed well using a glass stick and a vortexer. Centrifuge at 4°C for 20 min at 10,000 × g, discard the supernatant, collect the precipitate, and resuspend it in 1 mL PBS.
(e) The final collected IgY was placed in a dialysis bag (10 kDa) and dialyzed overnight with 0.1 % saline, then transferred to PBS dialysis for 4 h. The dialyzed samples were collected and stored at −20°C. The concentration of IgY was determined using a BCA protein quantification kit, and the purity of IgY was assessed by Sodium dodecyl sulfate polyacrylamide gel electrophoresis (12 % SDS-PAGE).

### 2.4 Determination of purified IgY's titer

The purified IgY's titer was tested using an indirect ELISA (iELISA) (checkerboard titration method). The following specific steps were taken: the *B. abortus* S66 antigen, fusion protein and LPS were diluted in a gradient of 1:400, 1:800, 1:1600, 1:3200, 1:6400, 1:12800, 1:25600, 1:51200, 1:102,400, 1:204800, 1:409600, and 1:819,200, starting from an initial concentration derived from immunized chickens. These dilutions were subsequently added to a 96-well plate (NUNC, Denmark) at a volume of 100 μL per well, spanning columns 1 to 12 overnight at 4°C. The next day, the plate was washed three times with PBST for 3 min each time. Then 5 % skimmed milk powder (300 μL/well) was added and incubated for 2 h at 37°C. The plate was again washed three times with PBST for 3 min each time. Three purified IgY (*Brucella*-IgY, Fusion protein-IgY and LPS-IgY, initial dilution 1:4000, doubled to 1:256,000) was then added (100 μL/well, Row A to G) and incubated at 37°C for 1 h, respectively. The Row H was PBS added as a negative control. After three further washes with PBST for 3 min each, HRP Conjugated Goat Anti-Chicken IgY (1:5000) (Thermo Fisher Scientific, USA) was added (100 μL/well) and incubated at 37°C for 1 h. The plate was then washed three times for 3 min each time with PBST. 3,3′,5,5′ Tetramethylbenzidine (TMB) substrate solution was added (100 μL/well) and allowed to react for 15 min at room temperature, protected from light. Finally, 50 μL/well stop solution was added and OD_450_ was measured using an ELISA VersaMax microplate reader (MD, USA). The antigen dilution with the ratio of the absorbance of the positive and negative (P/N) ≥2.1 was selected as the detection limit while maintaining a specific antibody titer.

### 2.5 Limit of detection assessment (indirect ELISA)

The *B. abortus* S66 (10^7^ CFU/mL) antigens were used in a coating buffer (CBS, pH 9.6). Subsequently, 100 μL of the prepared antigens were added to each well of 96-well plates at a 1:1000 dilution, doubling the ratio, followed by overnight incubation at 4°C for coating. After washing three times with PBST (PBS containing 0.05% Tween-20), the plate was then blocked with 5% skimmed milk for 2h at 37°C. After three additional washes with PBST, three IgY antibodies were added at a 1:10000 dilution. The negative control utilized was PBS. The plate was then incubated at 37°C for 1 h. After three washes with PBST, the plate underwent incubation with HRP Conjugated Goat Anti-Chicken IgY at a 1:5000 dilution for 1 h at 37°C. Following another round of washing, 100 μL of TMB substrate solution was added to each well, and the reaction proceeded for 15 min. Finally, 50 μL of stop solution (2M H_2_SO_4_) was added to halt the reaction. Optical density values were measured at 450 nm using VERSA max microplate reader (Molecular Devices Corp., Sunnyvale, CA, USA). The OD_450_ value was read using an enzyme marker. The results of the color reaction were expressed as the ratio of the OD_450_ of the positive and negative control (P/N). A criterion of P/N ≥ 2.1 was used for positive evaluations. Concurrently, IgG was also tested (HRP Conjugated Rabbit Anti-Bovine IgG was utilized for comparison).

### 2.6 Cross-reactivity assessment

An iELISA (checkerboard titration method) was utilized to assess the cross-reactivity of IgY as the primary antibody in the assay. The iELISA steps were same as described in Section 2.4, and the difference is *E. coli* O157:H7 (1 × 10^9^ CFU/mL), *E. coli* lysate (8.35 × 10^9^ CFU/mL), *O. anthropi* (1 × 10^9^ CFU/mL), *Salmonella* (1 × 10^9^ CFU/mL), *L. monocytogenes* (1.65 × 10^9^ CFU/mL), and *S. aureus* (3.85 × 10^9^ CFU/mL) as antigens were diluted with CBS at various concentrations, including 1:500, 1:1000, 1:2000, 1:4000, 1:8000, 1:16000, 1:32000, 1:64000, 1:128000, 1:256000, respectively, then each antigen was added to a 96-well plate (100 μL/well) overnight at 4°C. *B. abortus* S66 (1 × 10^9^ CFU/mL) served as the positive control. Positive results of cross-reactivity were determined by a P/N value ≥ 2.1. In comparison, IgG was run simultaneously as a control (utilizing HRP Conjugated Rabbit Anti-Bovine IgG).

### 2.7 Detection of simulation samples

Testing of simulated samples was conducted according to references (19, 20). Cow's milk and goat's milk were selected as food matrices to simulate food contamination scenarios from clinical, veterinary, and biosafety perspectives. To prepare food samples contaminated with *Brucella* spp., low-temperature pasteurized milk (3.8–4.5 % fat, purchased from a local supermarket) was obtained and swiftly transported to the laboratory at a low temperature within 15 min. The liquid samples were filtered using a 0.2 μm filter to eliminate solid residues and then mixed with PBS (1 % Tween 20 and 10 % FBS) at a 1:1 (w/v) ratio. *Brucella* was introduced into the milk samples to achieve final bacterial concentrations of 1 × 10^4^, 1 × 10^3^, 1 × 10^2^, 1 × 10^1^, and 1 × 10^0^ CFU/mL by spiking them with a known quantity of the bacteria to simulate contamination scenarios. These samples were preserved at low temperatures for further analysis.

Food samples were obtained from local markets and promptly transferred to the laboratory under low-temperature conditions within a 15 min timeframe. For the preparation of solid food simulation samples, samples were collected from beef, beef liver, lamb, goat liver, pork, and pig liver. 5 g of each sample were weighed, thoroughly rinsed with ultrapure water, ground using a grinder, and then autoclaved at 121°C for 20 min after confirming the absence of natural *Brucella* contamination using the national standard culture method (GB 18646-2018). Subsequently, 1 g of sterilized solid samples was taken, and 10 mL of sterilized physiological saline was added and mixed well to create the sample matrix. *Brucella* at varying concentrations was then inoculated into the matrix to achieve final bacterial concentrations of 1 × 10^4^, 1 × 10^3^, 1 × 10^2^, 1 × 10^1^, and 1 × 10^0^ CFU/mL, followed by storage at low temperatures for further analysis.

The aforementioned food simulation samples were subjected to a sandwich ELISA. In each well of 96-well plates, 100 μL of IgG against *B. abortus* diluted at 1:4000 was added, followed by an overnight incubation at 4°C. Subsequently, 100 μL of the simulation samples were added, and various types of IgY prepared in this study were utilized as the primary antibody at a dilution of 1:8000. HRP Conjugated Goat Anti-Chicken IgY was employed as the secondary antibody at a dilution of 1:5000. After each step, the wells were washed thrice with PBST. Each experimental set was conducted in triplicate, with PBS serving as the negative control.

### 2.8 Statistical methods

In this study, rigorous statistical methods were employed to ensure the validity and reproducibility of the findings. For comparisons among multiple groups (IgG, *Brucella*-IgY, Fusion protein-IgY, and LPS-IgY), one-way ANOVA (Welch) was used to assess overall differences. All statistical analyses were performed using GraphPad Prism 10 (GraphPad Prism10, USA), with a significance level set at *P* < 0.05. In the ELISA assays, a criterion of S/N ≥ 2.1 was used for positive evaluations, and regression analysis was performed to determine detection thresholds, with *R*^2^ values reported to assess the goodness of fit.

## 3 Results

### 3.1 Determination of IgY purity of yolk antibody

**Figure 1:**
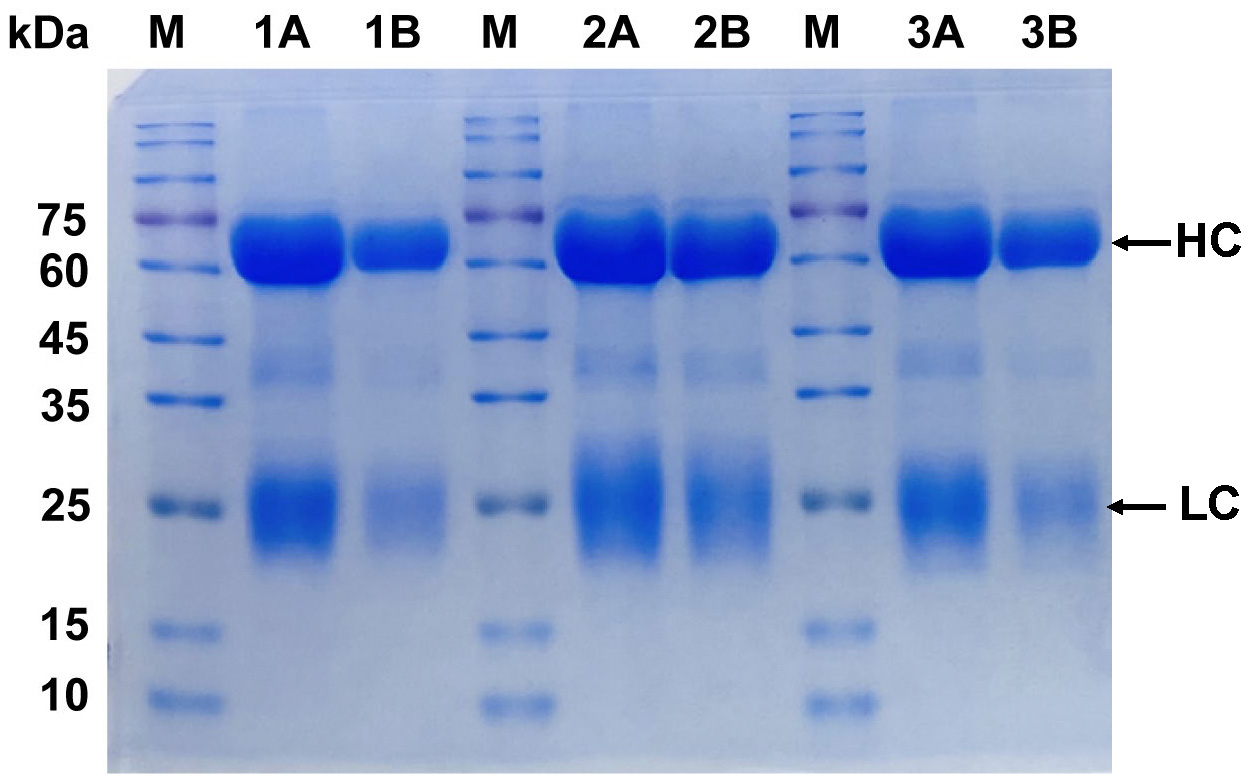
Purity analysis of IgY by SDS-PAGE. Lane 1A and 1B: *Brucella*-IgY; Lane 2A and 2B: Fusion protein-IgY; Lane 3A and 3B: LPS-IgY. M: Protein marker. The SDS-PAGE analysis shows high purity of the purified IgY antibodies, with heavy chains (HC) at 65–70 kDa and light chains (LC) at 22–30 kDa.

IgY was purified using the PEG6000 method and SDS-PAGE electrophoresis. It was demonstrated a high level of IgY purity (Figure 1). The target protein was observed to be cleaved into two chains as a result of disulfide bond breakage. The heavy chain (HC) exhibited a molecular weight range of 60–70 kDa, while the light chain (LC) displayed a molecular weight range of 25–30 kDa, indicating that the six IgY antibodies possessed similar levels of purity. The protein bands observed in the gel confirm the high purity of the purified specific IgY, with both the HC and LC clearly visible and devoid of structural damage.

### 3.2 Determination of IgY concentration

The standard curve of IgY concentration determined by using the BCA test kit is shown in Supplementary Figure S1. The curve equation is *y* = 0.6931*x* + 0.2231, *R* = 0.9988 0.99, and the standard curve conforms to the experimental requirements. From the standard curve, it can be seen that the IgY protein concentration of each group 1A:29. 776 mg/mL, 1B:25.910 mg/mL, 2A:30.786 mg/mL, 2B:28.709 mg/mL, 3A:25.304 mg/mL, 3B:30.382 mg/mL.

### 3.3 Determination of IgY antibody titer

**Figure 2:**
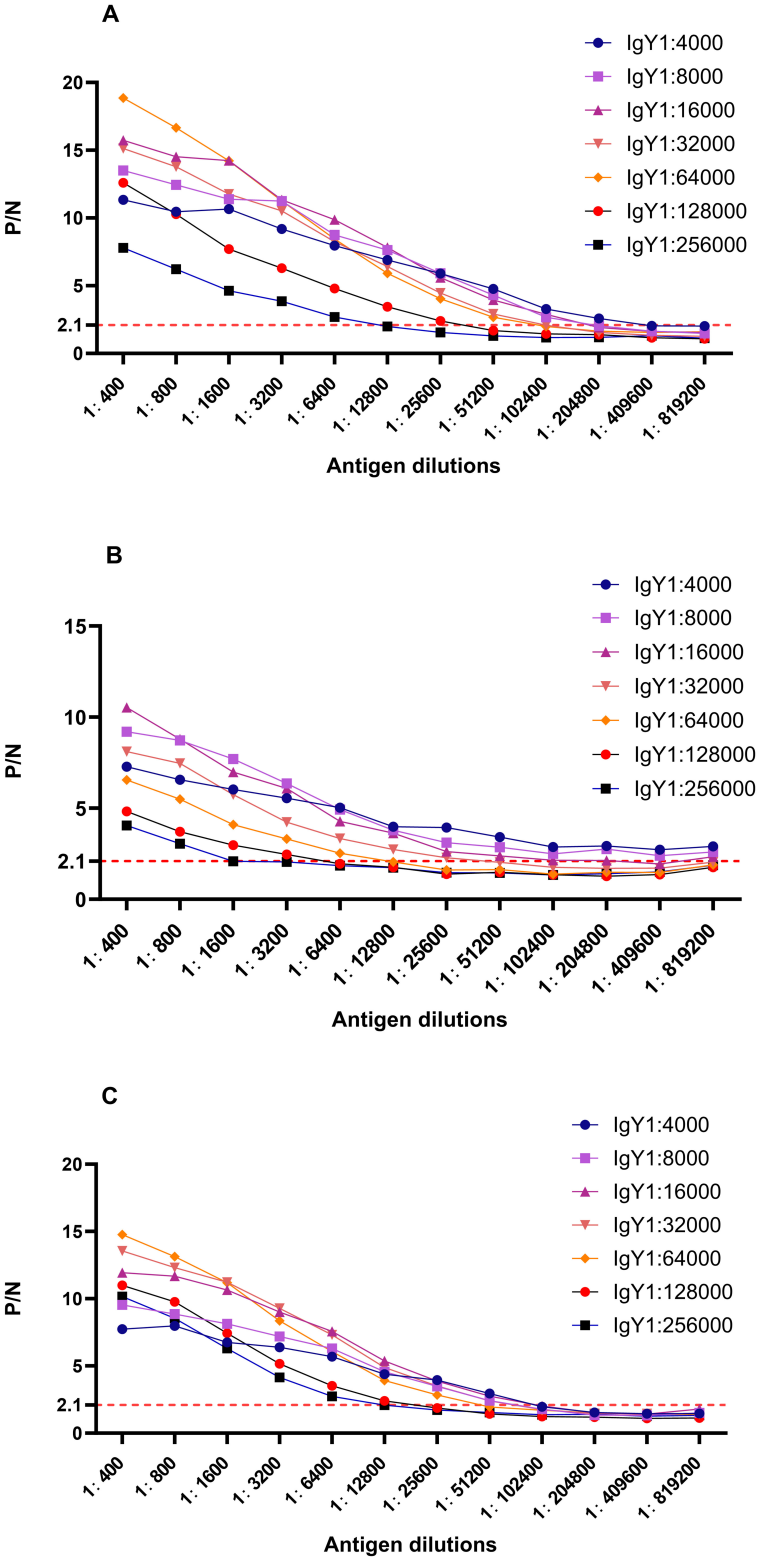
P/N curves for different dilutions of antigen and antibody. **(A)** *Brucella*-IgY; **(B)** Fusion protein -IgY; **(C)** LPS -IgY. The P/N values indicate the ratio of OD_450_ in test wells to that in negative control wells. The optimal dilutions for each IgY antibody were determined based on the highest P/N ratios, with *Brucella*-IgY showing the highest sensitivity.

Figure 2 illustrates the ratio curves of OD_450_ **(P)** and OD_450_ **(N)** in the test wells relative to the negative control wells at various antigen and antibody dilutions. Pertaining to *Brucella*-IgY, the P/N values surpassed those of the other curves at titers of 1:4000, 1:8000, 1:16000, 1:32000, and 1:64000 for the primary antibody IgY at the dilutions of *Brucella* were 1:204800, 1:102400, 1:102400, 1:102400, and 1:51200. As for fusion protein-IgY, the P/N values outperformed the other curves when the primary antibody IgY titers were 1:4000, 1:8000, 1:16000, and 1:32000. In these four curves, the dilutions of fusion protein were 1:819200, 1:819200, 1:204800, and 1:25600, respectively. Regarding LPS-IgY, the P/N values exceeded those of the other curves at primary antibody titers of 1:8000, 1:16000, 1:32000, and 1:64000. In these four curves, the dilutions of LPS were 1:51200, 1:51200, 1:51200, and 1:25600, respectively. Given the consistent trend in P/N value changes and the smooth curve observed at primary antibody titers of 1:4000 and 1:8000, the optimal concentration for the primary antibody IgY was determined to be 1:8000, taking into account the conservation of biological materials. Based on the experimental outcomes, it is evident that *Brucella*-IgY exhibited the highest titer in the assay. Conversely, the antibody titer of LPS-IgY surpassed that of fusion protein-IgY, indicating a higher titer of LPS-IgY in the assay compared to fusion protein-IgY.

### 3.4 Limit of detection and cross-reactivity comparison between IgY and IgG

**Figure 3:**
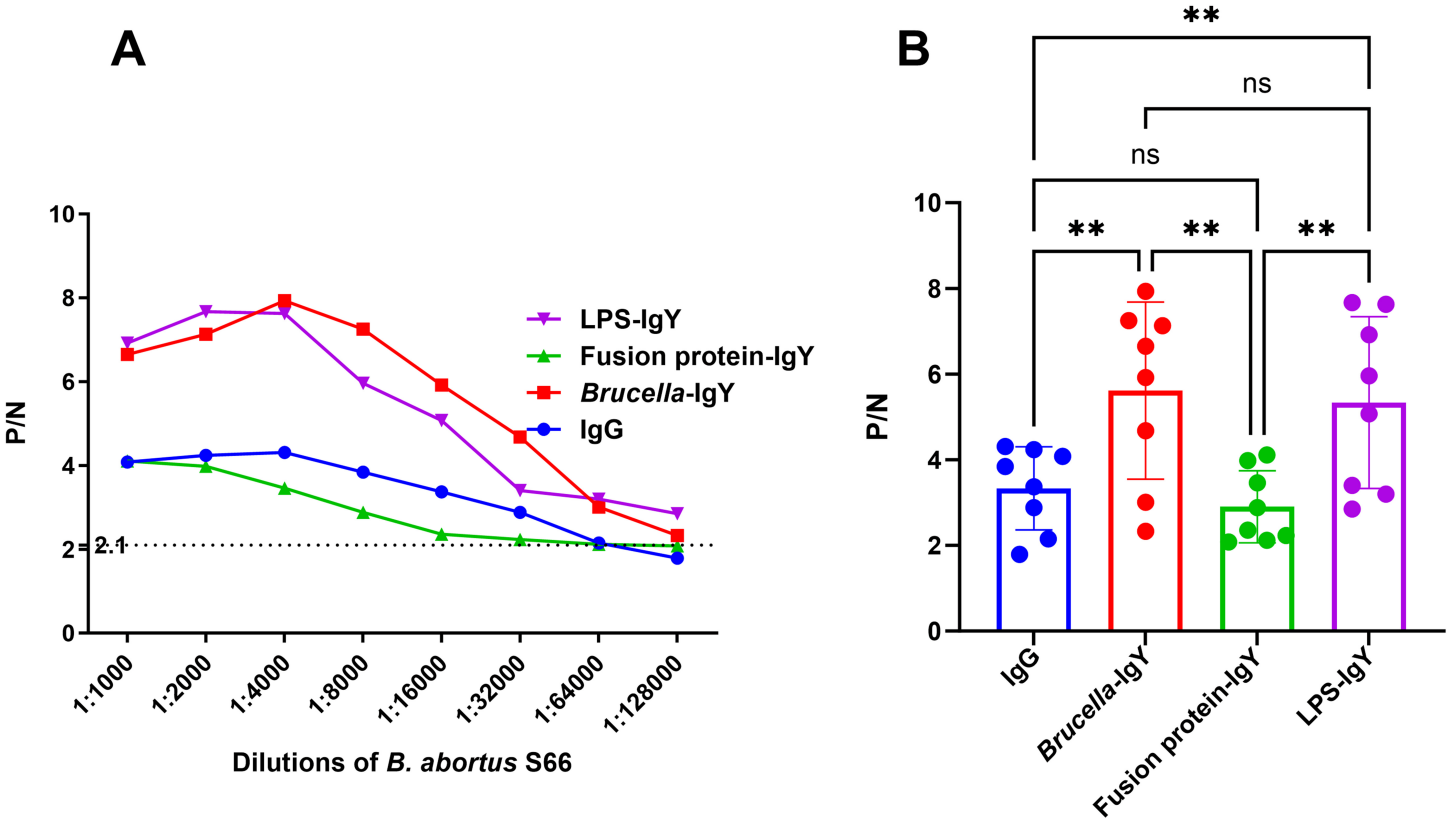
Comparison of limit of detection for IgY and IgG. The P/N values were plotted against various antigen dilutions. **(A)** P/N curves for different dilutions of *B. abortus* S66 antigen with various antibodies. The criterion for positive detection was set at S/N ≥ 2.1 (dotted line). **(B)** Relative levels of different antibodies compared using Welch ANOVA. Significant differences are indicated as **(*P* < 0.01) and ns (not significant).

In Figure 3, it is evident that the P/N values exhibited varying trends at different antibody dilutions; however, LPS-IgY and *Brucella*-IgY antibody curves surpassed those of IgG, indicating a potentially higher affinity of IgY antibodies toward *Brucella* antigens. The results indicated that when detecting *B. abortus*, positive antibody detection could still be achieved at a dilution of 1:64,000, suggesting that the use of *Brucella*-IgY and LPS-IgY antibodies resulted in a more sensitive detection limit for *B. abortus*.

Cross-reactivity assessment revealed that *Brucella*-IgY exhibited cross-reactivity with *E. coli* O157:H7 and *O. anthropi* under 1:2000 of antigen dilution and 1:16000 of IgY dilution, *E. coli* lysate under 1:4000 of antigen dilution and 1:32000 of IgY dilution, and *Salmonella* under 1:1000 of antigen dilution and 1:8000 of IgY dilutions. Fusion protein-IgY exhibited cross-reactivity with *E. coli* O157:H7 under 1:2000 of antigen dilution and 1:8000 of IgY dilution, *O. anthropi* under 1:32000 of antigen dilution and 1:32000 of IgY dilution, *E. coli* lysate under 1:8000 of antigen dilution and 1:32000 of IgY dilution, and *Salmonella* under 1:4000 of antigen dilution and 1:32000 of IgY dilutions. In contrast, IgG exhibited only minor cross-reactivity with *S. aureus* at 1:1000 of antigen dilutions, while LPS-IgY demonstrated lower cross-reactivity by failing to recognize interfering bacteria even when the concentration of bacterial fluids was increased unless at the 1:4000 dilution of IgY (Raw data is shown in Supplementary material S2). This highlights the lower cross-reactivity of LPS-IgY.

### 3.5 Food simulated samples testing

**Figure 4:**
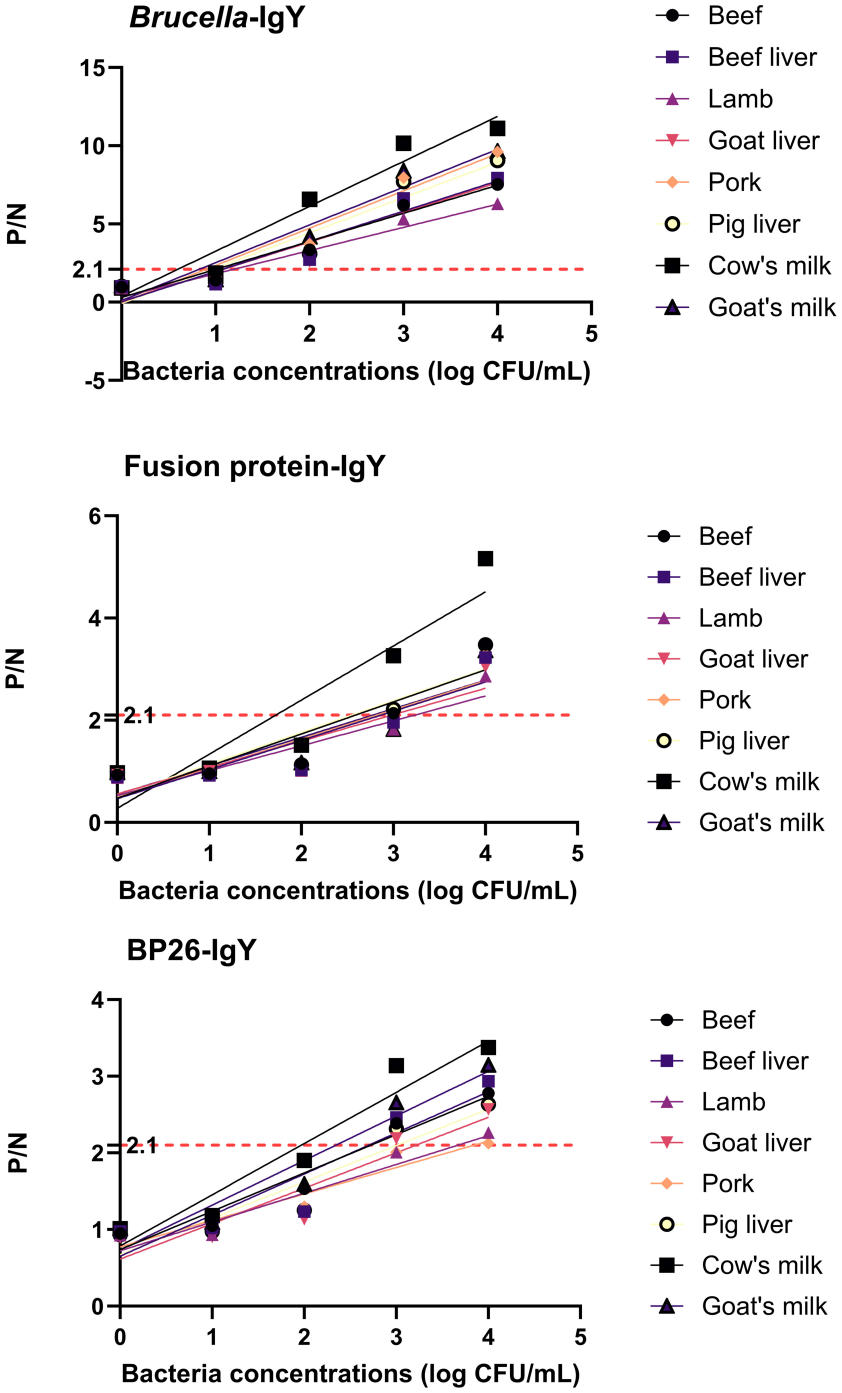
Test results of simulated samples. The figure shows the results of sandwich ELISA for detecting Brucella abortus in various food matrices (cow's milk, goat's milk, beef, beef liver, lamb, goat liver, pork, and pig liver). The P/N values were plotted for different bacterial concentrations (1 × 104 to 1 × 10° CFU/mL). The IgY antibodies demonstrated high sensitivity and specificity in detecting Brucella abortus, even at low concentrations. The results confirm the practical applicability of IgY antibodies in food safety testing.

*Brucella* detection in food-contaminated simulated samples was conducted using a sandwich ELISA without the need for any enrichment or processing steps. The mean values from three parallel experiments were used for comparison. Figure 4 and Table 1 illustrate that *B. abortus* was detectable at lower concentrations in simulated samples when utilizing IgG and IgY antibodies.

**Table 1:** Detection threshold of simulated samples using IgY antibodies.

**IgY**	**Sample**	**Regression equation**	***R* ^2^**	**P/N = 2.1 (log CFU/mL)**
*Brucella*-IgY	Beef	*y* = 1.792*x* + 0.3014	0.9563	1.004
	Beef liver	*y* = 1.930*x* + 0.02971	0.9115	1.073
	Lamb	*y* = 1.483*x* + 0.3321	0.9462	1.192
	Goat liver	*y* = 1.899*x* + 0.04613	0.9109	1.082
	Pork	*y* = 2.384*x*−0.03873	0.9375	0.897
	Pig liver	*y* = 2.255*x*−0.08156	0.9135	0.967
	Cow's milk	*y* = 2.871*x* + 0.3845	0.9499	0.598
	Goat's milk	*y* = 2.416*x* + 0.1056	0.9402	0.825
Fusion protein-IgY	Beef	*y* = 0.6275*x* + 0.4763	0.8202	2.588
	Beef liver	*y* = 0.5706*x* + 0.4658	0.8016	2.864
	Lamb	*y* = 0.4868*x* + 0.5272	0.8125	3.231
	Goat liver	*y* = 0.5167*x* + 0.5588	0.8065	2.983
	Pork	*y* = 0.5710*x* + 0.4906	0.7961	2.819
	Pig liver	*y* = 0.6216*x* + 0.5181	0.8148	2.545
	Cow's milk	*y* = 1.058*x* + 0.2775	0.8614	1.723
	Goat's milk	*y* = 0.5583*x* + 0.5498	0.7673	2.777
LPS-IgY	Beef	*y* = 0.4996*x* + 0.7392	0.9447	2.724
	Beef liver	*y* = 0.5365*x* + 0.6571	0.8693	2.689
	Lamb	*y* = 0.3754*x* + 0.7227	0.9023	3.669
	Goat liver	*y* = 0.4626*x* + 0.6138	0.8703	3.213
	Pork	*y* = 0.3453*x* + 0.7730	0.9148	3.843
	Pig liver	*y* = 0.4747*x* + 0.6717	0.8880	3.009
	Cow's milk	*y* = 0.6685*x* + 0.7845	0.9365	1.968
	Goat's milk	*y* = 0.5821*x* + 0.7360	0.9287	2.343

The table shows the regression equations, R^2^ values, and detection limits (P/N = 2.1) for *Brucella*-IgY, Fusion protein-IgY, and LPS-IgY in various food matrices. The results demonstrate the high sensitivity and specificity of IgY antibodies in detecting *Brucella* in food samples.

## 4 Discussion

IgY antibodies can be found in chicken blood and egg yolk. Numerous studies have shown the potential of IgY for immunotherapy and immunodiagnosis (21). The development of IgY-based immunoassays is an area that warrants further research, as IgY antibodies have been utilized in various techniques such as ELISA, protein blotting, immunohistochemistry, and immunochromatography (15, 22, 23). Our results indicate that immunization against *Brucella* can stimulate the chicken's immune system, leading to the production of IgY antibodies in eggs. The IgY method offers several advantages, including the non-invasive collection of antibodies from egg yolks, eliminating the need for blood collection and animal sacrifice. A small amount of antigen is required to achieve high and long-lasting IgY levels in immunized egg yolks, aligning with previous research findings (16, 24). Therefore, the production of IgY antibodies from immunized laying hens presents a cost-effective and rapid approach for developing preventive immunization strategies and immune detection methods without the need for invasive techniques.

Previous studies have highlighted the limitations of LPS-based immunological assays, such as poor limit of detection and cross-reactivity with various other Gram-negative bacteria (25). To enhance the limit of detection in this technique, specific IgY antibodies were developed for *Brucella* detection using *B. abortus* S66, fusion protein and LPS antigen. The fusion protein, comprising conserved epitopes from *Brucella*, was selected as the immunogen for IgY antibody development, the chosen epitopes are highly conserved among *Brucella* spp. but have low homology with other common bacteria, thereby reducing cross-reactivity and improving the specificity of the antibody for *Brucella* detection. In comparison to the other two IgY antibodies, the LPS-IgY generated in this study exhibited superior limit of detection and low cross-reactivity. LPS, a crucial antigenic component in *Brucella* (26), enables LPS-IgY to bind more effectively to *Brucella*-associated molecules, diminishing the assay's cross-reactivity. The abundance of LPS in the bacterial cell wall enhances the binding capability of LPS-IgY to target antigens, thereby improving the limit of detection. Notably, the fully immunized and screened LPS-IgY antibodies produced in this study exhibit higher affinity and tighter binding to the antigen, facilitating the detection of low concentrations of the target antigen and ultimately increasing the limit of detection.

The study results revealed that antibody levels in chicken serum were detectable at 14 days post-immunization, with a subsequent increase and sustained elevation after 42 days of immunization. Both *Brucella*-IgY and fusion protein-IgY achieved a potency of 1:640,000 by day 42. In contrast, LPS-IgY did not reach this level until day 56 post-immunization. Optimal collection of IgY antibodies from egg yolks was found to be between days 42 and 56 post-immunization, balancing the considerations of limit of detection and cost-effectiveness. *Brucella*-IgY exhibited the lowest detection limit in the assessment, while LPS-IgY demonstrated higher limit of detection and cross-reactivity in the assay. Importantly, IgY antibodies displayed low cross-reactivity with major foodborne pathogens. The low cross-reactivity of IgY antibodies toward *E. coli, O. anthropic, Salmonella*, and *S. aureus* can be attributed to the high specificity of the IgY antibodies generated against conserved *Brucella* epitopes. These epitopes are unique to *Brucella* and do not share significant homology with antigens from other bacteria. Additionally, the optimized detection threshold ensured that the antibodies only bound to their specific targets, minimizing cross-reactivity. This highlights the high specificity and selectivity of the IgY antibodies used in this study.

In conclusion, the use of IgY antibodies proved to be effective in accurately detecting *Brucella* in food samples. The results from the food simulation samples confirmed that IgY is a practical and promising tool for *Brucella* detection in milk or meat samples, exhibiting good limit of detection and low cross-reactivity. It was observed that the detection efficiency in meat samples using IgY antibodies was slightly lower compared to milk samples, possibly due to the presence of hemoglobin affecting the assay background and the interference of various proteins in meat samples. Nonetheless, a significant distinction was still observed between a weak positive result (*Brucella* concentration of 1,000 CFU/mL) and a negative result in the meat sample assay. This underscores the utility of the three IgY antibodies as primary detection agents for identifying low concentrations of target bacteria within complex food matrices. These findings highlight the efficacy of the IgY antibodies developed in this study for *Brucella* immunological detection.

In this study, it was observed that IgY antibodies exhibited a higher limit of detection compared to bovine IgG antibodies in the detection of *B. abortus*. This difference can be attributed to the structural dissimilarities between IgY and IgG antibodies, particularly in the Fc region where IgY antibodies have weaker binding to mammalian Fc receptors, thereby reducing nonspecific background signals (27, 28). As a result, the use of IgY antibodies may offer enhanced sensitivity in detecting target molecules or antigens in specific experimental settings. Overall, the developed IgY antibodies demonstrated promising potential for applications in *Brucella* research and related experiments, proving to be effective tools for immunological detection of *Brucella*. However, IgY is still unable to overcome some of the shortcomings of serology, such as the inability to rapidly identify *Brucella* spp. as PCR can. The study assessed its cross-reactivity by selecting a limited number of pathogenic organisms from a vast array of foodborne pathogenic microorganisms, notably excluding *Yersinia enterocolitica*. Further investigation into the cross-reactivity is warranted in future research.

## Data Availability

The original contributions presented in the study are included in the article/Supplementary material, further inquiries can be directed to the corresponding authors.

## Supplementary material

Supplementary material S1 Details for the preparation of antigens.

Supplementary material S2 Raw data for iELISA and sandwich ELISA.
