# Supplementary material for: Preparation and application of specific chicken yolk antibodies in detecting Brucella
Source: Front Vet Sci. 2025 May 12;12:1552097. doi: 10.3389/fvets.2025.1552097 (PMC12104676; doi:10.3389/fvets.2025.1552097)
Supplement: Supplementary material S1 — Details for the preparation of antigens. [file Data_Sheet_1.docx]

**Preparation and application of specific chicken yolk antibodies in** **detecting *Brucella***

**Xinru Qi^1^**^†^**, Yun Wang^2^**^†^**^*^, Qianhan Huang^1^**^†^**, Shiqi Zhao^1^, Qichuan Pei^1^, Yixiao Chen^1^, Dehui Yin^1,3*^, Tiansong Zhan^1,3*^**

1. Jiangsu Engineering Research Center of Biological Data Mining and Healthcare Transformation, Xuzhou Medical University, Xuzhou, Jiangsu, 221004, China

2. Department of Dermatology, the Affiliated Huai'an Hospital of Xuzhou Medical University, the Second People's Hospital of Huai'an, Huai'an, 223002, China

3. Key Laboratory of Human Genetics and Environmental Medicine, Xuzhou Medical University, Xuzhou, Jiangsu, 221004, China

† These authors contributed equally to this work

* Corresponding author:

**Yun Wang**, Department of Dermatology, the Affiliated Huai'an Hospital of Xuzhou Medical University & The Second People's Hospital of Huai'an, Huai'an, 223002, China. E-mail: wangyun5782@163.com

**Dehui Yin**, School of Public Health, Xuzhou Medical University, Xuzhou, 221004, China. E-mail: yindh16@xzhmu.edu.cn

**Tiansong Zhan,** School of Public Health, Xuzhou Medical University, Xuzhou, 221004, China. E-mail: tszhan@xzhmu.edu.cn

**1. P****reparation of antigens**

**1.1 Preparation of LPS antigen**

The LPS from the *B. abortus* A19 vaccine strain was extracted using the phenol-water method. Subsequently, the *B. abortus* A19 vaccine strain was cultured in TSB medium, followed by centrifugation to collect the organisms, which were then resuspended in PBS and heated to 66°C. Simultaneously, a 90 % phenol aqueous solution was heated to the same temperature. The two components were combined and subjected to centrifugation at 4°C. The resulting supernatant, containing LPS, was left to stand at room temperature for 24 h. The phenol phase (bottom layer) was then recovered and filtered. To the crude extracted LPS, 1 % saturated sodium acetate in methanol was added at three times the volume of LPS, and the mixture was left to precipitate at 4°C for 2 h. After collection, the precipitate underwent another round of centrifugation before the supernatant was retained. The remaining precipitate was dissolved in sterilized purified water, subjected to centrifugation, and the supernatant was collected. This supernatant was combined with the previously retained supernatant, filtered, and treated with trichloroacetic acid. The resulting mixture was centrifuged, and the supernatant was dialyzed, lyophilized, weighed, and prepared for use.

**1.2 Preparation of bacteria antigens**

1.2.1 Bacterial Expansion Culture

Use a sterile inoculation loop to take the preserved bacterial strain and inoculate it into 500 mL of LB medium (for *Escherichia coli* O157: H7, *Listeria monocytogenes*) or TSB medium (for *Salmonella*, *Staphylococcus aureus*, *B. abortus* S66 and *Ochrobactrum anthropic*). Incubate at 37°C with shaking for 24-48 h. Take 1 mL for bacterial counting; the remaining medium is treated with 1 % formaldehyde to inactivate the bacteria and stored at 4°C for later use.

1.2.2 Bacterial Counting (Plate Dilution Method)

Take 8 sterile 15 mL tubes, adding 9 mL of physiological saline to each. Label them sequentially from 1 to 8. Add 1 mL of the expanded culture (before inactivation) to tube 1, mix thoroughly, then transfer 1 mL of the mixed solution to tube 2. Continue this process to perform 10-fold serial dilutions. For tubes 5 to 8, take 1 mL of the diluted solution and evenly spread it onto LA or TSA agar plates. Incubate at 37°C for 24-48 h, with 3 replicate plates for each dilution. Observe and count the colonies. Based on the counting results, calculate the concentration of bacteria in the expanded culture medium. Centrifuge the original inactivated culture at 1500 xg to collect the bacteria, adjust the bacterial concentration to 10^9^ CFU/mL with physiological saline, and store at -20°C.

All the above operations are performed in a biosafety level 3(BSL-3) laboratory.

**2. Supplementary Figures and tables**

**Figure S1. Standard curve for concentration of protein of BCA method**


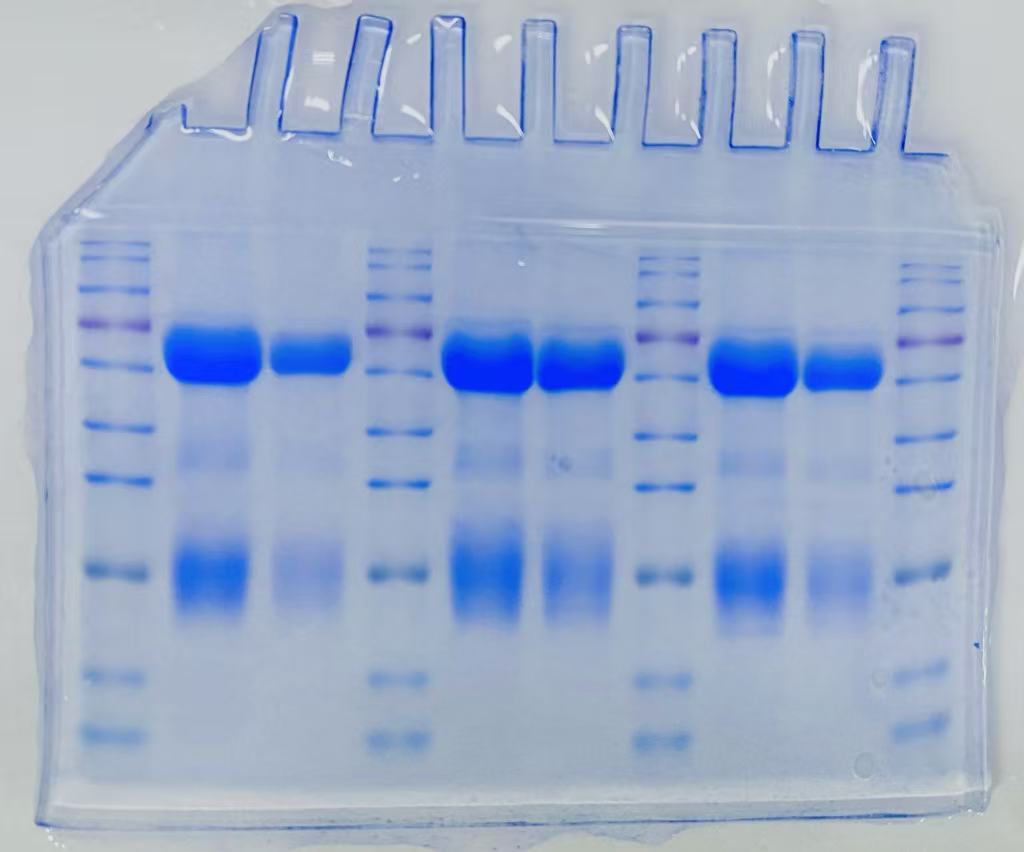


**Figure S2. Original image of SDS-PAGE analysis of purified IgY.**
